# Proteogenomic discovery of sORF-encoded peptides associated with bacterial virulence in *Yersinia pestis*

**DOI:** 10.1038/s42003-021-02759-x

**Published:** 2021-11-02

**Authors:** Shiyang Cao, Xinyue Liu, Yin Huang, Yanfeng Yan, Congli Zhou, Chen Shao, Ruifu Yang, Weimin Zhu, Zongmin Du, Chenxi Jia

**Affiliations:** 1 State Key Laboratory of Pathogen and Biosecurity, Beijing Institute of Microbiology and Epidemiology, 100071 Beijing, China; 2 State Key Laboratory of Proteomics, Beijing Proteome Research Center, Beijing Institute of Lifeomics, National Center for Protein Sciences (The PHOENIX Center, Beijing), 102206 Beijing, China; 3 School of Life Sciences, Hebei University, Hebei Province 071002 Baoding, China

**Keywords:** Proteomic analysis, Pathogens, Proteomics, Peptides

## Abstract

Plague caused by *Yersinia pestis* is one of the deadliest diseases. However, many molecular mechanisms of bacterial virulence remain unclear. This study engaged in the discovery of small open reading frame (sORF)-encoded peptides (SEPs) in *Y. pestis*. An integrated proteogenomic pipeline was established, and an atlas containing 76 SEPs was described. Bioinformatic analysis indicated that 20% of these SEPs were secreted or localized to the transmembrane and that 33% contained functional domains. Two SEPs, named SEPs-yp1 and -yp2 and encoded in noncoding regions, were selected by comparative peptidomics analysis under host-specific environments and high-salinity stress. They displayed important roles in the regulation of antiphagocytic capability in a thorough functional assay. Remarkable attenuation of virulence in mice was observed in the SEP-deleted mutants. Further global proteomic analysis indicated that SEPs-yp1 and -yp2 affected the bacterial metabolic pathways, and SEP-yp1 was associated with the bacterial virulence by modulating the expression of key virulence factors of the *Yersinia* type III secretion system. Our study provides a rich resource for research on *Y. pestis* and plague, and the findings on SEP-yp1 and SEP-yp2 shed light on the molecular mechanism of bacterial virulence.

## Abstract (web summary)

Shiyang Cao, Xinyue Liu, Yin Huang, and Yanfeng Yan et al. utilized an integrated proteogenomic approach to describe an atlas of small open reading frame-encoded peptides (SEPs) in the pathogen, *Yersinia pestis*. They demonstrate that two of these SEPs are associated with regulation of bacterial virulence, and altogether develop a valuable resource for future research into *Y. pestis* physiology.

## Introduction

Plague represents one of the most lethal diseases ravaging humans throughout history, for example, in the three historical pandemics: Justinian Plague, the Black Death and the Modern Plague^1–3^. The gram-negative bacterium *Yersinia pestis* is the plague pathogen mainly transmitted by fleas in rodents and humans as well as many other animal hosts^4^. *Yersinia pestis* shows excellent resilience and survival against the host immune system due to its unique genome and biological features, causing a fulminant disease. In the modern world, the bacterium survives in sylvatic animal reservoirs, often resulting in endemic plague outbreaks, which are still a potential threat to biosafety and public health^2^. However, many underlying mechanisms of bacterial infection and spread are still unclear. One of the reasons is the lack of accurate knowledge of the functional molecules in bacteria. Understanding these molecular mechanisms will help to develop more efficient strategies for plague treatment and prevention measures.

Emerging evidence indicates that small open-reading frame (sORF)-encoded peptides (SEPs) play important roles in the biological processes of bacteria^5–8^, which either act as signaling factors by binding to receptors or assist other regulatory proteins or complexes in exerting functions^9^. These SEPs were reported to be involved in various biological processes of bacteria, including stress sensing (Prli42), spore formation (SpoVM and CmpA), cell division (MciZ, SidA and Blr), transport of ions and macromolecules (KdpF, AcrZ and SgrT), kinases and signal transduction (MgrB and Sda), and also act as membrane-bound enzymes (CydX, PmrR and MgtR) and chaperones (MntS, FbpB and FbpC)^5,6^. In addition to these nonsecreted SEPs, some SEPs were reported to be secreted from bacteria, regulating the quorum-sensing (AgrD) and phage lysis/lysogeny decisions (AimPs)^6^. However, these intriguing molecules were previously overlooked due to biased annotation and limited discovery approaches. Usually, ORFs containing fewer than 100 codons are excluded by most conventional genome annotation tools. For instance, the submission system of GenBank filters out sequences with lengths <200 nucleotides^5^. Additionally, because of their small size and unique molecular features, SEPs are easily lost during sample preparation and analysis in the traditional proteomic workflows. Recently, Miravet-Verde et al. developed the bioinformatics tool RanSEPs and predicted 109 bacterial small ORFomes^10^. Sberro et al. conducted a comparative genomics study on 1773 human-associated metagenomes and predicted more than 4000 small protein families^11^. Inspired by these findings from large-scale bacterial genomic analysis, we hypothesize that there are many hidden SEPs in *Y. pestis* playing regulatory roles.

In this study, we established a proteogenomic pipeline for the identification and functional characterization of SEPs in *Y. pestis*. Three modules were integrated into this pipeline, including parallel SEP prediction from RNA-sequencing (RNA-seq) data, discovery by refined peptidomics approaches, and curation based on homolog search and spectrum inspection, resulting in an atlas of 76 *Y. pestis* SEPs. Among them, two virulence-regulatory SEPs, SEP-yp1 and SEP-yp2, were identified by thorough functional characterization and encoded by sequences previously known as noncoding regions. They play important roles in the regulation of the antiphagocytic capability and virulence of *Y. pestis*. Remarkably, SEP-yp1 modulated the expression of the key components in the *Yersinia* type III secretion system (T3SS). Collectively, the description of the SEP atlas opens new avenues for research on *Y. pestis* and plague, and characterization of the two virulence-regulatory SEPs provides insights into the underlying mechanism of the bacterial pathogenicity.

## Results

### An integrated proteogenomic pipeline enables the discovery of SEPs in *Y. pestis*

**Fig. 1:**
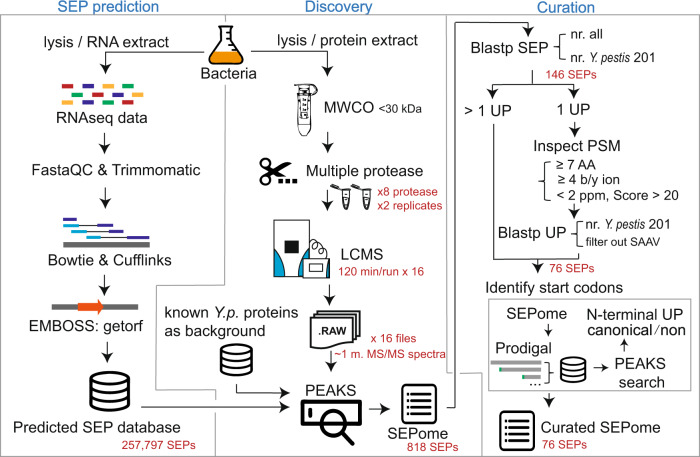
The integrated workflow for discovery and identification of SEPs in *Y. pestis*. It includes three modules: prediction, discovery, and curation. In the prediction module, the RNA-seq data are processed and assembled to generate a searchable SEP database. In the discovery module, the bacteria samples were prepared by a refined peptidomics approach, respectively, with eight proteases, followed by LC-MS analysis. The MS raw data were searched by PEAKS engine against the predicted SEP database with a known protein database as search background. In the curation module, the resulting SEPome was processed by BLASTP to remove the known proteins and then filtered at the level of peptide-spectrum match (PSM), and then the N terminus was determined to generate the final SEPome. Refer to Supplementary Data 1 and 2 for the details of SEP candidates. MWCO molecular weight cut-off, UP unique peptide, AA amino acid, SAAV single amino acid variant.

A human-avirulent *Y. pestis* strain *biovar Microtus* 201 isolated from the rodent Brandt’s vole (*Microtus brandti*)^12^, which is lethal to rodents, was used in this study. The genome of this strain consists of one chromosome and four plasmids, pPCP1, pCD1, pMT1, and pCRY^12^. Our integrated pipeline for the discovery of the bacterial SEPs includes three modules: prediction, discovery, and curation (Fig. 1).

In the prediction module, we conducted RNA-seq analysis of *Y. pestis* strain 201 grown under various stress conditions to achieve the greatest diversity of transcripts. The transcript templates were obtained by the conventional sequential processing of the RNA-seq reads, i.e., quality control by using FastaQC and Trimmomatic as well as alignment and assembly by using Bowtie^13^ and Cufflinks^14^. Next, a three-reading-frame translation was performed with *Y. pestis* transcripts from the upstream stop codon to the immediate downstream stop codon, as reported^13,14^, by using getorf from EMBOSS, resulting in a SEP database of 257,797 sequences at lengths ranging from 8 to 150 amino acids. This approach allows retaining the longest sequence for translation and avoids misidentification of upstream start codons.

In the discovery module, we adopted a strategy reported by Saghatelian and colleagues^15,16^ for enriching SEPs <30 kDa. To improve the sequencing coverage of SEPs, we employed eight enzymes (trypsin, LysC, LysN, GluC, AspN, ArgC, chymotrypsin, and mirror trypsin) in eight independent experiments (two biological replicates each) for ultradeep analysis of the bacterial extract. In total, 16 liquid chromatography-mass spectrometry (LC-MS) raw data, including nearly one million tandem mass spectra, were collected and further subjected to a PEAKS search against the predicted SEP database. Note that the fragments of known proteins could also pass the 30 kDa molecular weight cut-off filter and be coeluted with the SEPs during sample preparation, causing false-positive identification. To exclude them, we collected *Y. pestis* proteins from UniProt as the background database for the PEAKS search. We obtained 818 SEP candidates with a false discovery rate (FDR) of 0.01 (Supplementary Data 1).

In the curation module, the exactly matched SEPs to known proteins were first removed from the list through a BLASTP search. The remaining SEPs were grouped into two categories: one belongs to putative proteins for all bacterial species and the other is specific for *Y. pestis biovar Microtus* 201. Following the application of the rigorous criteria evaluated by Miravet-Verde et al.^10^, the remaining SEP candidates with more than two unique peptides were considered confident identifications. To expand the identification list, the SEP candidates with one unique peptide were further evaluated by inspecting the peptide-spectrum matches of unique peptides under the four criteria: (i) amino acid number ≥7, (ii) concessive by ions ≥4, (iii) parent ion <2 parts per million (p.p.m.), and (iv) PEAKS score >20. Only those that passed the criteria were further subjected to a BLASTP search against the *Y. pestis* 201 nr. database to filter out single amino acid variants as well as fragments of known proteins. Finally, 76 SEPs were obtained (Supplementary Data 1 lists the SEPs at every curation step). Among them, 55 SEPs were encoded by chromosomes, and 4, 5, 10, and 2 SEPs were encoded in the pCD1, pCRY, pMT1, and pPCP1 plasmids, respectively.

Because of heterogeneous translational initiation of bacterial genes, we further sought to identify translation initiation sites and possible alternative translation sites of these 76 SEPs by using an approach reported by Wang et al.^17^ and a prokaryote-specific algorithm, Prodigal^18^. The resulting SEP sequences with predicted translation initiation sites were compiled into a database against which a further search of 16 LC-MS raw data was conducted. In total, 56 unique N-terminal peptides corresponding to 18 canonical translation initiation sites were identified. Among them, 11 unique peptides for translation initiation sites were obtained corresponding to three downstream N termini of the canonical translation initiation sites (Supplementary Data 2 lists the N-terminus information of SEPs).

### A large proportion of these *Y. pestis* SEPs are predicted to be functional

**Fig. 2:**
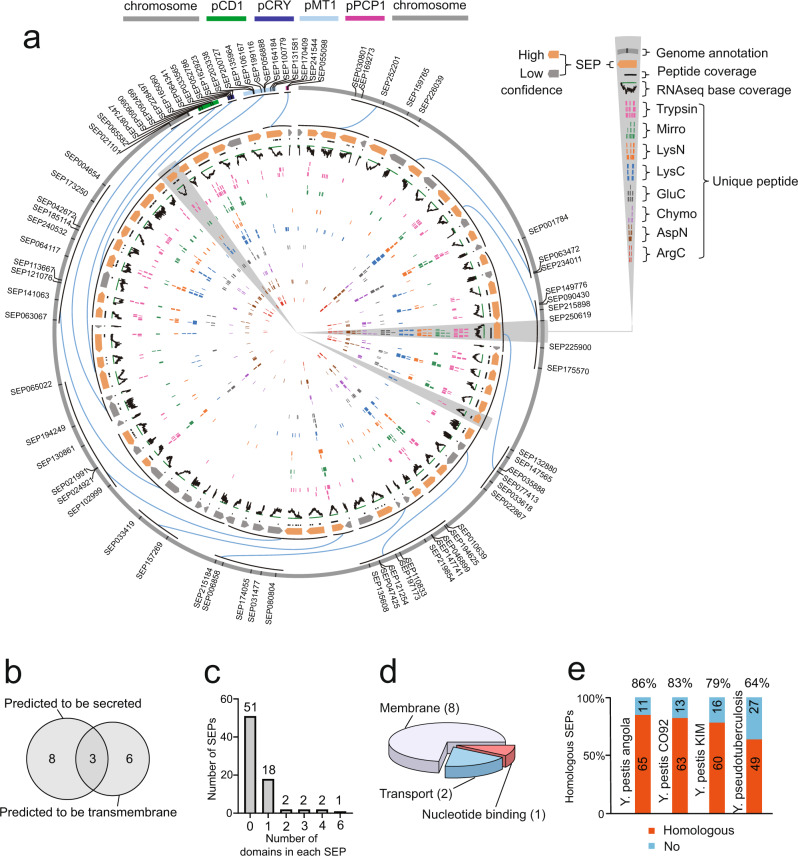
The atlas of identified SEPome. **a** Circos plot showing the genome, transcription and protein information of the identified 76 SEPs. The Circos labels were listed in the upper right of the panel. Briefly, the information of unique peptides, RNA-seq base coverage, and genome annotation were shown from inside to outside rings. The outermost ring represents the chromosome and four plasmids (information labeled in the color bands at the top of the panel). Unique peptides resulting from eight proteases were, respectively, in eight colors. Each unique peptide maps the amino acid sequence position on the SEPs. Identifications with one unique peptide after manual curation represent low confidence, and greater than one unique peptide is of high confidence. The RNA-seq reads coverage were generated by alignment of reads to nucleotide position. Refer to Supplementary Data 1 for the details of the SEPs. **b** Venn diagram showing secreted and transmembrane proteins as predicted by SignalP and TMHMM among the 76 SEPs. **c** Distribution of domain-containing SEPs as predicted by Pfam among the 76 SEPs. **d** Functional inference among the 76 SEPs as determined using BLASTP against the SEP dataset from a previous report^10^. **e** Homologous analysis of the 76 SEPs using tblastn in human-virulent bacterial strains. The homologous SEPs are selected by identities >90% and *e* value <1e^−3^. The detailed results of the bioinformatic analysis are listed in Supplementary Data 3. Mirror mirror trypsin.

To provide a bird’s-eye view of the identified SEPs, we constructed a Circos plot (Fig. 2a and details shown in Supplementary Data 1) that shows genomic origins, transcriptomic base coverage, and unique peptide coverage resulting from different protease digestions (from outside to inside rings of the Circos plot). The benefit of using different protease digestions was demonstrated by the improved identification frequency (Supplementary Fig. 1a) and sequence coverage (Supplementary Fig. 1b) of SEPs.

Generally, bacterial pathogens interact with host cells by direct cell–cell contact or via small diffusible molecules secreted by cells. We employed TMHMM^19^ and SignalP^20^ to investigate the two-dimensional structure of these SEPs and found that 11 of them were predicted to be transmembrane and 9 SEPs were secreted, including 3 SEPs that were both transmembrane and secreted (Fig. 2b and Supplementary Data 3). An alternative algorithm, Phobius^21^, was used for prediction, and an additional seven-transmembrane and five secreted SEPs were obtained (Supplementary Data 3).

Protein domains provide valuable insights into the function, and many distantly related organisms share evolutionally conserved domains that have similar functions. We next performed a domain search on the 76 identified SEPs against the Pfam database^22^. Approximately 33% of them were matched to Pfam domains, and seven SEPs contained more than two domains in the sequences (Fig. 2c), suggesting that they were probably functionally active.

Next, we performed functional annotation on these SEPs by using a sequence homology search. Most recently, Miravet-Verde et al.^10^ reported 109 bacterial sORFomes by combining RanSEPs with omics approaches. Among them, 5175 SEPs were NCBI annotated and associated with functions. We compiled the 5175 function-annotated SEPs into a database and ran a BLAST homolog search against this database on our 76 identified SEPs. Eleven of them were matched into the database with a functional inference, including membrane, transport, nucleotide binding, etc. (Fig. 2d and Supplementary Data 3). Interestingly, three SEPs (SEP046899, SEP064341, and SEP132880) were annotated in the membrane category and were also predicted to contain transmembrane sequences (Supplementary Data 3).

Subsequently, we investigated whether these identified SEPs are conserved in human-virulent *Y. pestis* strains, including *Y. pestis* angola^23^, *Y. pestis* CO92^24^, *Y. pestis* KIM^25^, and *Y. pseudotuberculosis* strain NCTC10275. The results from the tblastn search indicate that more than 79% of these 76 SEPs are conserved in the three human-virulent *Y. pestis* strains, and 64% of them are conserved in *Y. pseudotuberculosis* (Fig. 2e and Supplementary Data 3). The high conservation rate indicates that investigation of the functional roles of these SEPs is important for understanding the pathogenicity of the plague to humans.

### Comparative peptidomics analysis under host-specific environments and high-salinity stress identifies functional SEP candidates

**Fig. 3:**
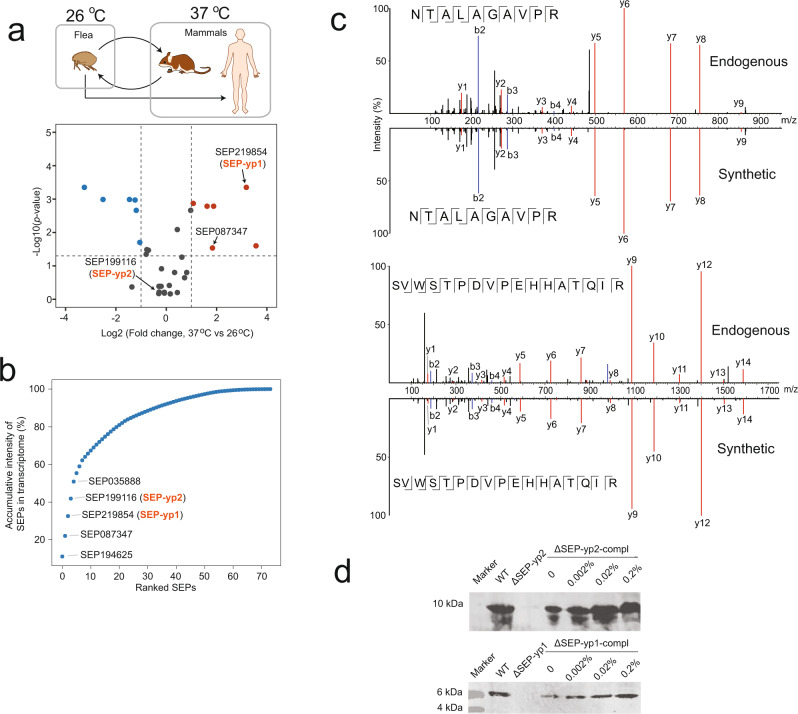
Functional screening and molecular validation of the identified SEPs. **a** Quantitative peptidomics analysis of SEPs between two in vitro conditions mimicking the flea vector and rodents’ temperatures. The upper panel shows the transmission of bacteria between flea and mammal hosts as well as the in vitro experimental conditions. The lower panel displays a volcano plot of quantitative results of SEPs between the two conditions. The *p* value was calculated by unpaired *t* test and adjusted with Benjamini–Hochberg procedure. Refer to Supplementary Data 4 for details. **b** Accumulative intensity of the ranked 76 identified SEPs in the transcriptome. The top five most abundance of SEPs are labeled in the panel. Refer to Supplementary Data 4 for details. **c** Mirror plot showing the tandem mass spectra of endogenous and synthetic peptides, respectively, from SEP-yp1 (top panel) and SEP-yp2 (bottom panel). The b and y ions are, respectively, labeled with blue and red. **d** Western blotting of SEP-yp2 and SEP-yp1 showing bacterial lysis of *Y. pestis* WT, ΔSEP and ΔSEP-compl. Arabinose was added in the ΔSEP-compl strains at the concentrations of 0, 0.02, 0.2, and 2% to induce the expression of SEPs.

Intrigued by the fact that a large proportion of these SEPs were predicted to be functional, we performed label-free quantitative peptidomics experiments to screen the functional SEP candidates that are potentially associated with the virulence of the plague. *Yersinia pestis* is primarily transmitted among wild rodents via flea bite^2^. The typical body temperatures of flea vectors and rodents are 26 and 37 °C, respectively. By culturing the bacteria under the two temperatures, we were able to mimic the two typical niches of *Y. pestis* in vitro (the top panel of Fig. 3a) to compare the expression levels of SEPs between the two temperatures. Thirty-two SEPs were quantifiable by using the trypsin digestion procedure, as summarized in a volcano plot (the bottom panel of Fig. 3a, details are listed in Supplementary Data 4). The expression level of SEP219854 (named SEP-yp1) was increased in the bacteria cultured at 37 °C. These results suggest that the expression of SEP-yp1 was much higher at the mammalian body temperature and possibly plays a role in adaptation to the adverse host environments during plague infection.

*Yersinia pestis* requires rapid and adaptive responses to salinity stress during transmission and infection^26,27^. Therefore, we explored the abundance changes of the 76 identified SEPs in the high-salinity response. The results of comparative analysis between the bacterial samples grown with and without 2.5% NaCl were summarized into a volcano plot (Supplementary Fig. 2 and Supplementary Data 4). SEP199116 (named as SEP-yp2) was downregulated under high-salinity stress, suggesting a possible role of SEP-yp2 in response to this adverse stimulus. The accumulative intensities of transcripts of the 76 SEPs were summarized into a ranked plot (Fig. 3b and Supplementary Data 4), and the top five SEPs, including SEP-yp1 and SEP-yp2, occupied almost 50% of the total intensity. In addition, the two SEPs are encoded by sequences previously known as noncoding regions. SEP-yp1 is encoded by a downstream sequence of YP_1841^12^ and is expressed with the same orientation of the gene. SEP-yp2 is encoded by an intergenic sequence between genes *pMT70* and *pMT71*^12^ and is expressed with the same orientation as *pMT71*. In addition, the two SEPs showed 100% of sequence identities in the three human-virulent strains, including angola, CO92 and KIM. We thus chose SEP-yp1 and SEP-yp2 for further functional analysis.

### The presence of SEP-yp1 and SEP-yp2 was confirmed by synthetic peptide standards and Western blotting

To validate the correctness of the sequences of SEP-yp1 and SEP-yp2, their tryptic peptides were chemically synthesized and analyzed by LC-MS. The tandem mass spectra of synthetic and endogenous peptides were compared and their fragment ions were exactly matched (Fig. 3c). In addition, SEP-yp2 was determined to be initiated from the first AUG codon (Supplementary Data 2), while the start codon of SEP-yp1 was not successfully identified. To further determine this, acidified methanol was used to directly extract the endogenous peptides from bacteria followed by LC-MS analysis without proteolytic cleavage. Three endogenous peptide fragments were detected by confident sequence matches (Supplementary Fig. 3), which rigorously determined the AUG start codons of SEP-yp2 and SEP-yp1.

Subsequently, mouse monoclonal antibodies against SEP-yp1 and rabbit polyclonal antibodies against SEP-yp2 were prepared and used for Western blotting on bacterial extracts. Two bands with sizes of approximately 9 kDa and 5 kDa were detected by the two antibodies (Fig. 3d). The two bands disappeared when the two SEPs were deleted, demonstrating the presence of endogenous SEP-yp1 and SEP-yp2. The expression levels of the two SEPs were gradually elevated in the mutants complemented with the plasmids expressing SEP-yp1 and SEP-yp2 when the bacterial strains were grown at gradient concentrations of arabinose that induced the expression of the corresponding genes. Taken together, these MS and Western blotting results confirmed the existence of SEP-yp1 and SEP-yp2 in *Y. pestis*.

### Deletion of SEP-yp1 and SEP-yp2 attenuates the intracellular survival capability of *Y. pestis*

**Fig. 4:**
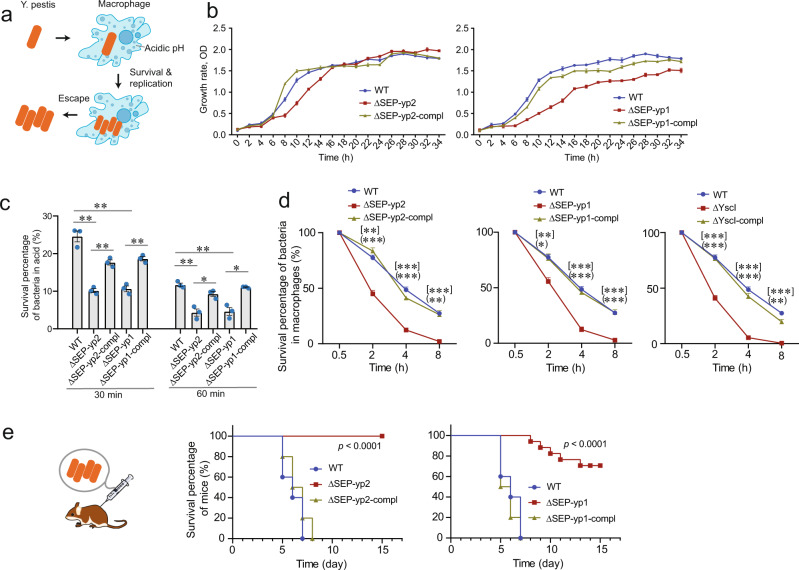
Phenotype screening of SEP-yp1 and SEP-yp2 by a comparative assessment of WT, ΔSEP, and ΔSEP-compl bacterial strains. **a** Schematic showing the interaction of *Y. pestis* with macrophages. The bacteria enter macrophages and develop antiphagocytic capability, followed by escape and transmission. **b** Plot of bacterial growth rate showing the influence of SEP deletion on bacterial proliferation and growth. *N* = 3 in each group. Mean ± SEM. **c** Plot of bacterial survival percentage in acidic environment showing the capability of acidic resistance effected by SEP deletion. *N* = 3 in each group. Unpaired *t* test. Mean ± SEM. **d** Plot of bacterial survival percentage in macrophages affected by SEP deletion. *N* = 3 in each group. Mean ± SEM. Unpaired *t* test. The *p* values of ΔSEP vs. WT are shown in [] at each time point, and ΔSEP-compl vs. ΔSEP in (). For panels **c** and **d**, *p value < 0.05, **p value < 0.01, and ***p value < 0.001. **e** Animal survival curve showing the bacterial virulence by subcutaneous injection to mice. WT, *N* = 10; ΔSEP, *N* = 20; and ΔSEP-compl, *N* = 10. A log-rank test was used to calculate the statistical significance. Note that some error bars are too small to be visualized.

During the early phase of infection, *Y. pestis* efficiently adapts itself to the host niche in the macrophages and soon develops resistance to phagocytosis, followed by an escape from the skin to lymph nodes (Fig. 4a). This antiphagocytic ability is essential for *Y. pestis* to establish a systemic infection^4,28^. Herein, we examined the phenotypic changes in antiphagocytic capabilities after the deletion of SEP-yp1 and SEP-yp2. First, successful survival in macrophages requires the normal proliferation of pathogens to overcome the host elimination. In an in vitro culture assay, we observed that deletion of SEP-yp1 or SEP-yp2 resulted in a decreased growth rate and that expression of SEP-yp1 or SEP-yp2 in the mutant strains restored the phenotype of normal growth (Fig. 4b). Second, the pH values of the macrophage compartment of mammalian hosts range from 4.5 to 6.2, which acts as a host defense to clear the engulfed bacteria^29^. By measuring the bacterial survival at acidic pH values in vitro, we observed that deletion of SEP-yp1 or SEP-yp2 reduced the acid survivals of bacteria, and a successful rescue was then achieved (Fig. 4c). Third, the intracellular survival capability of bacteria was examined. We found that deletion of SEP-yp1 or SEP-yp2 caused almost no loss of bacterial survival in macrophages after 4 h of infection, and the intracellular survival capability was recovered to normal in the complemented strains (Fig. 4d). Taken together, these results indicate that both SEP-yp1 and SEP-yp2 have important regulatory functions in the physiological processes and the intracellular survival capability of *Y. pestis*.

### Deletion of SEP-yp1 and SEP-yp2 attenuates the virulence of *Y. pestis* in mice

*Yersinia pestis* virulence to animals is the ultimate phenotype for its pathogenicity. Therefore, we evaluated the effect of SEP-yp1 and SEP-yp2 on the virulence of *Y. pestis* with a mouse infection model. Our previous study shows that the LD_50_ value of the wild-type *Y. pestis* strain 201 is 3 colony-forming unit (CFU)^30^. In this study, each animal was challenged by subcutaneous infection with 100 CFU of either wild-type or mutant *Y. pestis* (>30-fold LD_50_). The death of wild-type-infected animals was observed on the fifth day, and all succumbed at 8 days post infection (Fig. 4e and Supplementary Fig. 4). In strong contrast, all the mice challenged with the SEP-yp2 mutants survived in the 2-week experiment. For those challenged with the SEP-yp1 mutant, the death of mice occurred on the eighth day post infection, and 75% of mice survived after 2 weeks. The virulence effect can be fully recovered in the complemented strains. These results indicated that SEP-yp1 and SEP-yp2 are intrinsic peptides that play critical roles in the pathogenicity of *Y. pestis*.

### Quantitative proteomics reveals downregulation of virulence factors after deletion of SEP-yp1

**Fig. 5:**
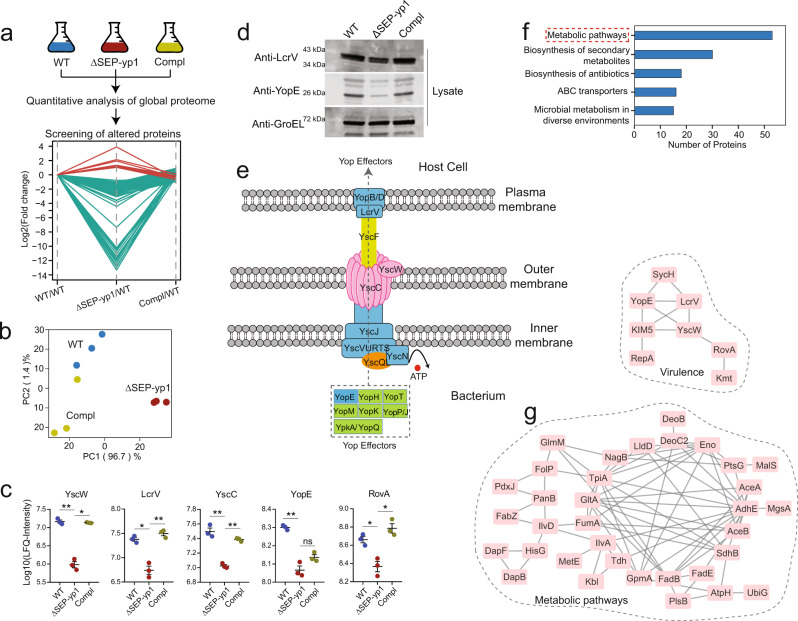
Quantitative global proteome analysis of WT, ΔSEP-yp1, and ΔSEP-yp1-compl (compl) reveals that SEP-yp1 deletion reduces the expression of virulence proteins. **a** Experimental workflow of label-free quantitative proteomics for screening the altered proteins in the bacteria due to SEP-yp1 deletion. *N* = 3 in each group. The multiple line plot shows the 189 proteins filtered by the criteria: opposite alteration trends between ΔSEP-yp1/WT and Compl/ΔSEP-yp1, *p* value (ΔSEP-yp1/WT) < 0.05, *p* value (Compl/ΔSEP-yp1) < 0.05, and *p* value (Compl/WT) > 0.05. **b** PCA analysis of all the quantifiable proteins in the three groups. **c** Scatter plots show the alteration of four altered virulence factors as well as RovA by quantitative proteomics. The statistical significance was calculated by unpaired *t* test adjusted by Benjamini–Hochberg procedure with FDR < 0.01. *P value < 0.05 and **p value < 0.01; ns nonsignificant. Error bars represent SEM. **d** Orthogonal validation of altered proteins by Western blotting. Each experiment has three biological replicates. **e** Schematic representation of *Yersinia* type III secretion apparatus. **f** KEGG analysis of the 189 altered proteins due to SEP-yp1 knockout. **g** Mapping the altered proteins onto the protein interaction network using the STRING database. One cluster represents the proteins involved in the metabolic pathways from KEGG analysis and another is associated with bacterial virulence. Refer to Supplementary Data 5 for details.

Intrigued by the remarkable phenotypic changes of the SEP-yp1 and SEP-yp2 on bacterial virulence in mice, we sought to collect evidence at the molecular level to explore their potential virulence-regulatory roles. Label-free quantitative analysis of the bacterial proteome under mammalian host-specific conditions (37 °C) was carried out (Fig. 5a; note that the analysis procedures for SEP-yp1 and SEP-yp2 are the same, and only that of SEP-yp1 is shown). Specifically, wild-type *Y. pestis* (group named WT), together with SEP-yp1-deleted mutants (ΔSEP-yp1) and its complemented strain (ΔSEP-yp1-compl), was cultured under the mammalian host conditions at 37 °C. The bacterial pellets were collected and prepared by using a standard proteomic analysis procedure for one-shot LC-MS analysis with a 2-h gradient. PEAKS searching against a *Y. pestis* protein database (containing 4136 entries) resulted in 1146 quantifiable proteins (Supplementary Data 5), which were subjected to further bioinformatic analysis.

Principal component analysis (PCA) indicated that the nine samples were clustered into three groups, and the ΔSEP-yp1 group was well separated from the WT and ΔSEP-yp1-compl groups in the first component with 96.7% variability (Fig. 5b). Additionally, Pearson’s correlation analysis (Supplementary Fig. 5A) showed that the ΔSEP-yp1 group had relatively lower correlation coefficients with the WT and ΔSEP-yp1-compl groups. These results indicate that the proteome profile of the WT group is close to that of ΔSEP-yp1-compl and different from that of ΔSEP-yp1. Consistently, the ΔSEP-yp1 group displayed a different phenotype from the WT, which was recovered in the ΔSEP-yp1-compl group (Fig. 4b–e), suggesting an association between the proteome profile and the physiological phenotype.

As shown in the cluster plot of Fig. 5a and the heatmap of Supplementary Fig. 5B, the altered proteomic profile resulting from SEP-yp1 deletion, which was rescued in the ΔSEP-yp1-compl group, included 8 upregulated and 181 downregulated proteins (Supplementary Data 5). Remarkably, four virulence factors, LcrV, YscW, YscC, and YopE, were among the downregulated proteins in the ΔSEP-yp1 group, and their expression was rescued in the ΔSEP-yp1-compl group (Fig. 5c). Two of them were further orthogonally validated by Western blotting (Fig. 5d). In the T3SS responsible for the virulence of *Y. pestis*^31^, YscC belongs to a superfamily of bacterial outer membrane proteins existing as a stable oligomeric complex (Fig. 5e), essential for the secretion of anti-host factors. YscW lipoprotein modulates localization of YscC complex on outer membrane^32^. LcrV assists in the insertion of the pore-forming proteins into the host cell. Among the six *Yersinia* outer protein effector proteins, YopE is one of the highly translocated effectors into host cells and is essential for virulence^32^. The downregulation of these proteins in the injectisome and effectors by SEP-yp1 deletion suggests that SEP-yp1 plays a crucial regulatory role in the T3SS and bacterial virulence. Another important global regulatory factor, RovA, was downregulated (Fig. 5c).

Next, the 189 altered proteins in Fig. 5a were subjected to Kyoto Encyclopedia of Genes and Genomes (KEGG) and gene ontology (GO) analysis. A large fraction of proteins was linked to metabolic pathways (Fig. 5f) as well as metabolic processes (Supplementary Fig. 5c). Further protein–protein interaction analysis of the 189 altered proteins by the Search Tool for the Retrieval of Interacting Genes/Proteins (STRING) identified a protein cluster involved in metabolic pathways (Fig. 5g), consistent with the results of GO and KEGG analysis. The altered metabolic pathways are possibly associated with phenotypic changes in intracellular survival.

The proteomics data for SEP-yp2 were subjected to a similar analytical procedure as that of SEP-yp1 described above (Supplementary Data 6). Pearson’s correlation analysis (Supplementary Fig. 6a) and PCA (Supplementary Fig. 6b) suggested that the ΔSEP-yp2 group had a different proteome profile than the WT and ΔSEP-yp2-compl groups. The altered proteins were selected (Supplementary Fig. 6c) and subjected to GO and KEGG analysis, showing enrichment in metabolic processes (Supplementary Fig. 6d) and metabolic pathways (Supplementary Fig. 6e), respectively. Among the differentially expressed proteins resulting from deletion of SEP-yp1 and SEP-yp2, only 16 proteins are overlapped, with the remaining 93% were individually distributed (Supplementary Fig. 7), suggesting that SEP-yp1 and SEP-yp2 possibly execute their functions through different molecular mechanisms, which was also implied by the different expression patterns of the two SEPs in the host-specific environment (Fig. 3a) and high-salinity stress experiments (Supplementary Fig. 2).

### Deletion of SEP-yp1 influences the translocation of the T3SS

**Fig. 6:**
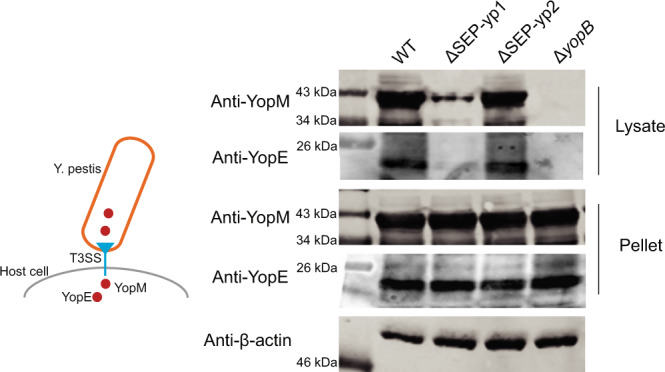
Deletion of SEP-yp1 influences the translocation of T3SS. HeLa cells were infected with *Y. pestis* strains for 2 h. After removing the media and thoroughly washing the wells, the infected cells were lysed by Triton X-100 buffer, since it is unable to break the cell wall of bacteria. The mixture was centrifuged to separate the lysate and the cell debris pellet. The samples were immunoblotted. Each assay has three biological replicates.

Subsequently, we examined whether the translocation efficiency of T3SS^33^ can be affected by SEP-yp1 and SEP-yp2. HeLa cells were, respectively, infected with the wild-type *Y. pestis*, the SEP-yp1-deleted mutant, or the SEP-yp2-deleted mutant. The *yopB*-deleted mutant was used as a negative control, since YopB is an essential protein for translocation without effecting the expression of T3SS^34^. After removing the culture medium, the buffer containing Triton X-100 was used for the lysis of HeLa cells, since it is unable to break the cell wall of bacteria. Subsequently, the injected amounts of YopE and YopM in the cell lysates were assayed (Fig. 6). YopM and YopE were detected in the cell debris pellets since the residual bacteria were present. In the lysates of the cells infected with the WT or SEP-yp2-deleted mutants, YopM and YopE were found to be present at comparable levels, indicating that T3SS translocation with deletion of SEP-yp2 was normal. In contrast, the abundances of YopM and YopE were decreased in the cells infected with the SEP-yp1-deleted mutant and, as expected, were unable to be detected in the cells infected with the *yopB*-deleted mutant, indicating that T3SS translocation was strongly inhibited by deletion of SEP-yp1. Therefore, we assumed that SEP-yp1 modulated the translocation efficiency of the T3SS.

## Discussion

*Yersinia pestis* is the pathogen of bubonic plague, among the deadliest human infectious diseases in history. However, many underlying mechanisms of bacterial infection and spreading remain unclear. Emerging evidence shows that the SEPs are important regulators in bacteria^5,6^. Here, we identified 76 *Y. pestis* SEPs with high confidence by using our robust integrated pipeline, which featured: (i) the use of eight proteases to improve the sequence coverage and identification confidence, (ii) a set of rigorous filters to remove false-positive identifications, (iii) the prediction of molecular structures and functions, and (iv) quantitative screening to identify functional candidates. This integrated pipeline can also be applied to other prokaryotic species for the discovery of these intriguing molecules. In contrast to previous works on prokaryotic SEPomes^10,11^, we carried out an extensive MS-based peptidomic investigation to obtain evidence at the protein level. Most of the 76 identified SEPs are conserved in the human-virulent *Y. pestis* strains as well as *Y. pseudotuberculosis*. Our SEP atlas provides a valuable resource for in-depth studies of *Y. pestis*.

The virulence-regulatory role of SEP-yp1 was strongly demonstrated by the feature of host-specific expression as well as the modulation of virulence in mice and T3SS. We observed that SEP-yp1 modulated the T3SS by regulating the expression of critical components, including YscW, LcrV, YscC, and YopE. The former three proteins are components of the needle-like T3SS injection isome, and the latter one is an effector. During infection of host cells, *Y. pestis* translocates effector proteins from the bacterial cytoplasm to the host cytosol by using the T3SS. Our results show that SEP-yp1 modulates not only the translocation of the T3SS but also the expression of effector proteins. In the experiments involving subcutaneous injection of bacteria into mice, deletion of SEP-yp1 elongated the survival time of animals. One of the reasons could be the inhibited expression of these virulence factors and altered function of T3SS by deletion of SEP-yp1. In addition to this attenuated effect on the T3SS, deletion of SEP-yp1 caused a slower growth rate, influenced metabolic pathways, and caused a large number of downregulated proteins in the bacteria, suggesting that some important functions in physiological processes were impaired. All of these factors might contribute to the phenotypic changes of the mutants in terms of virulence in mice as well as on acid resistance and intracellular survival. Another important feature of SEP-yp1 is its elevated expression under in vitro mammalian host conditions at 37 °C, suggesting that SEP-yp1 might act as a regulator during mammalian infection. The mechanism underlying how SEP-yp1 modulates virulence is worth further investigation.

We found that SEP-yp1 and SEP-yp2 share three common features. First, prediction of signal peptides and transmembrane sequences revealed that SEP-yp1 and SEP-yp2 do not contain any of these molecular features, suggesting their nonsecreted roles. Second, quantitative proteomics results indicate that deletion of SEPs inhibits metabolic pathways. Third, they exhibit wide effects on bacterial physiologies, including acid tolerance, growth rate, and virulence to mice. To elucidate the associations among these findings, we can find some clues from a review article by Duval and Cossart^6^, in which bacterial SEPs were sorted into two categories: secreted and nonsecreted. The secreted SEPs act as factors for communication or competition. In contrast, nonsecreted or cytoplasmic SEPs are involved in the cellular metabolism, or in sensing and responding to environmental changes. One example is the SgrT peptide translated from a small RNA that regulates the glucose–phosphate stress response^35^. These previous reports are consistent with our findings: SEP-yp1 and SEP-yp2 are nonsecreted and affect cellular metabolism, possibly further resulting in these bacterial phenotypes regarding acidic tolerance, growth, and virulence. To the best of our knowledge, SEP-yp1 and SEP-yp2 are the first reported SEPs with virulence-regulatory functions in *Y. pestis* thus far.

In conclusion, SEP-yp1 and SEP-yp2 are important regulators of bacterial virulence and metabolism and are associated with various bacterial physiologies. The data resource of the discovered *Y. pestis* SEPome is information-rich and demonstrates the power of our proteogenomic pipeline, painting a picture of these valuable molecules at the genome, transcriptome, and proteome levels. Further investigation of the nature of these previously overlooked molecules will shed light on many fundamental questions of *Y. pestis* and bubonic plague.

## Methods

### Bacterial strains and cell culture

The plasmids and bacterial strains used in this study were listed in Supplementary Table 1. *Yersinia pestis biovar Microtus* strain 201 is avirulent to humans, but highly virulent to mice^36^. *Yersinia pestis* strain 201 was cultured in Luria-Bertani broth at 26 °C or in a chemically defined TMH medium (with or without 2.5 mM calcium) at 26 °C or 37 °C^37^ and then 100 mg/mL ampicillin and 50 mg/mL kanamycin were added to the medium. RAW264.7 was maintained in Dulbecco’s modified Eagle’s medium (HyClone, Little Chalfont, UK) containing 10% fetal bovine serum at 37 °C in a 5% CO_2_ incubator.

### RNA-seq of *Y. pestis*

*Yersinia pestis* strain 201 were cultivated under various conditions to achieve the greatest diversity of messenger RNA transcripts, including 26 °C in TMH, 37 °C in TMH without CaCl_2_, 37 °C in TMH without CaCl_2_ (pH 6.0), 37 °C in TMH with 2.5% NaCl, 4 °C in TMH, and 42 °C in TMH in an incubator shaking at 200 r.p.m. overnight. An equal amount of bacterial cells (2.65 × 10^7^ CFU/mL) grown under the aforementioned conditions were mixed together, and the total RNA was then isolated for RNA-seq analysis. Libraries were prepared using the Illumina TruSeq Stranded Total RNA Kit according to the manufacturer’s protocol starting with 1 µg of total RNA. Sequencing with paired-end adaptors was performed on an Illumina HiSeq 2500 instrument by Novogene Co. Two biological replicates were performed with three technical replicates each.

### Prediction of SEPs from RNA-seq dataset and construction of the SEP database

The raw RNA-seq data were processed with FASTQC (v0.11.9) for quality check and then processed with Trimmomatic (v0.39)^38^ to remove the adapters and low-quality sequences. Bowtie2 (v2.2.0) software^13^ was used to align the high-quality clean reads against the genome of *Y. pestis*, which was downloaded in FASTA formats from the NCBI RefSeq website. The resulting binary sequencing files (*.bam) were processed by Cufflinks^14^ using FPKM normalization. The resulting assembly transcripts were translated in three frames to identify ORFs using the program getorf from the EMBOSS package (v6.4.0.0)^39^. We applied two filter criteria to keep protein-coding sORFs for SEP database construction for spectra identification: (i) set SEP of 8–150 amino acids in length and (ii) remove duplicate sequences.

### Sample preparation for identification of SEPs

For identification of SEPs in *Y. pestis*, the samples were prepared following the protocol reported by Slavoff et al.^16^. The bacteria were heated in boiling water for 10 min to denature the protease and then transferred on ice for sonication for 2 min with a 30% duty cycle. The bacteria lysis was centrifuged at 12,000 × *g* for 10 min at 4 °C. The supernatant was processed by a 30 kDa molecular weight cut-off filter. The flow-through samples were measured by bicinchoninic acid assay to evaluate the protein concentration and then tried down at low temperature in a SpeedVac vacuum concentrator. The pellets were resuspended in 8 M urea/50 mM Tris-HCl (pH 8) and reduced with 10 mM dithiothreitol at 56 °C for 30 min and alkylated with 10 mM iodoacetamide in the dark at room temperature for 30 min. After four times dilution with 50 mM Tris-HCl (pH 8), the solution was added with one of the eight enzymes at an enzyme to substrate ratio of 1:50, including trypsin, LysN, LysC, GluC, chymotrypsin, AspN, ArgC (Promega, USA), and mirror trypsin (Beijing Hua LiShi Scientific Co., China) in sequencing grade, as previously reported^17,40,41^. The digestion was performed overnight at 37 °C and quenched by adding 2 µL of formic acid. The digested peptides were then loaded on a C18 tip. After washing with water containing 0.1% formic acid, the peptide mixture was eluted by 50% acetonitrile containing 0.1% formic acid and then dried down for storage at a −80 °C refrigerator. Each enzyme experiment has two biological replicates.

### LC-MS analysis of SEP digests

The LC-MS experiments were performed on an Orbitrap Q-Exactive HF mass spectrometer (Thermo Scientific) coupled with an online Easy-nLC 1200 nano-HPLC system (Thermo Scientific). Five microliters of a sample containing 500 ng peptide mixture were loaded on a reversed-phase C18 trapping column (3 μm, 2 cm × 100 μm ID) and then separated on a nano-HPLC C18 column (1.9 μm, 12 cm × 150 μm ID) at a flow rate of 600 nL/min with a 120-min gradient: 4–8% solvent B for 10 min, 8–28% for 95 min, 28–40% for 11 min, 40–95% for 1 min, and 95% for 3 min (Solvent A, water; Solvent B, acetonitrile; 0.1% formic acid). The electrospray voltage was 2.2 kV. Peptides were analyzed by data-dependent tandem mass (MS/MS) acquisition mode with a resolution of 120,000 at full scan mode and 15,000 at MS/MS mode. The full scan was processed in the Orbitrap from *m*/*z* 250 to 1800; the top 20 most intense ions in each scan were automatically selected for higher-energy collisional dissociation (HCD) fragmentation with a normalized collision energy of 29% and measured in an Orbitrap. Typical mass spectrometric conditions were: automatic gain control targets were 3 × e^6^ ions for full scans and 5 × e^2^ for MS/MS scans; the maximum injection time was 80 ms for full scans and 80 ms for MS/MS scans; and dynamic exclusion was employed for 13 s.

### MS data processing and bioinformatic analysis of SEPs

The MS raw data were analyzed by PEAKS Studio v8.5 against the home-made *Y. pestis* SEPome database. A UniProt FASTA database containing 4605 proteins of *Y. pestis* (downloaded on August 21, 2017) was used as the contaminant database to filter out any peptide fragments of known proteins during searching. Mass tolerance was set to a maximum of 10 p.p.m. for peptide masses and 0.02 Da for HCD fragment ion masses. The enzyme was set to one of the proteases used for sample preparation, including trypsin, mirror trypsin, LysN, LysC, GluC, chymotrypsin, AspN, or ArgC. The oxidation (M) and acetylation (N-term) were set as variable modifications and carbamidomethylation (C) as fixed modifications. Up to three missed cleavages were allowed. The unique peptides were filtered by FDR < 1% at the peptide level. The label-free quantitation based on extracted ion chromatograms was performed using the PEAKS Q module. Only peptides with valid quantitative values ≥75% in at least one group were kept. The missing values were imputed with 10% of the minimum quantifiable intensities of the SEP. The peptide intensities were normalized by the total ion chromatogram. The quantitative information was exported as.csv files for further bioinformatics processing. The R scripts were used for statistical analysis and data visualization, including Circos plot, volcano plot, hierarchical clustering, PCA, etc.

### Functional prediction of SEPs using SignalP, TMHMM, Phobius, and Pfam

SingalP 5.0 was used to predict the signaling peptide of the identified 76 SEPs with gram-negative mode and default parameters^20^. TMHMM was used to predict the transmembrane sequence of SEPs with default parameters^19^. Phobius was also run with gram-negative mode and default parameters^21^. The online version of the hmmscan program^42^ was used to test the presence of conserved protein domains with the Pfam parameters^22^. We used BLASTP search against the database of 5175 bacterial SEPs previously reported^10^ to find homologs domain targets region using cut-off of *e* value <1e^−05^.

### Construction of the *Y. pestis* mutants and complemented strains

ΔSEP-yp1 and ΔSEP-yp2 were generated by replacing the coding sequences of SEP-yp1 and SEP-yp2 (1–186 bases) with the kanamycin resistance cassette via the λRed recombination system^43^. Only 1–186 bases of SEP-yp1 was replaced in order not to affect the function of SEP-yp1 downstream genes. To construct complemented strains, a PCR-generated DNA fragment containing the SEP-yp1 or SEP-yp2 coding sequences was cloned into plasmid pBAD24. Then, the recombinant plasmids were introduced into the mutants.

### SEP-yp1 and SEP-yp2 gene expression in *Y. pestis*

Wide-type *Y*. pestis, ΔSEP-yp1, ΔSEP-yp2, 201-pBAD24-SEP-yp1, and 201-pBAD24-SEP-yp2 were grown at 26 °C in TMH medium to OD_620 nm_ = 1.0. Equal amounts of proteins in pellets were analyzed by sodium dodecyl sulfate–polyacrylamide gel electrophoresis (SDS-PAGE) gel and immunoblotting with anti-SEP-yp2, YopE, and LcrV rabbit polyclonal antibodies and anti-SEP-yp1 mouse monoclonal antibody at a concentration of 1 µg/mL^44^. Among them, Mini-PROTEAN Tris-Tricine precast gels were used for the analysis of SEP-yp1 and SEP-yp2. Images of the immunoblotting results were acquired with the Odyssey SA imaging system (LI-CRO).

### Assessment of growth rate of *Y. pestis*

Wide-type *Y. pestis*, mutants, and complemented strains were grown in TMH medium at 26 °C overnight. Then, bacterial cultures were diluted 1:20 in a fresh TMH medium and incubated at 26 °C with shaking at 220 r.p.m. The bacterial growth was monitored every 2 h by measuring the absorbance of OD_620_^45^.

### Assessment of acid resistance of *Y. pestis*

Wide-type *Y. pestis*, mutants and complemented strains were grown in the TMH medium at 26 °C until OD_620_ = 1.0. Then, the bacterial cells were harvested and resuspended in *Y. pestis* 20 mM glucose minimal medium^46^. A 1:100 dilution of bacterial resuspension was added to pH 3.5 minimal medium as the experimental group or added to pH 6.5 minimal medium as a control. After incubation at room temperature for 30 min (and 60 min in a separate group), the bacterial suspensions were diluted and plated on a Hottinger’s agar to determine the number of viable bacteria in triplicate^47^.

### Assessment of survival capability of *Y. pestis* in macrophages

Wide-type *Y. pestis*, mutants, and complemented strains were grown in the TMH medium at 26 °C until OD_620 nm_ = 1.0. RAW264.7 were seeded into 24-well plates and incubated to reach about 90% confluency before infection. RAW264.7 were infected with wide-type *Y. pestis*, mutants, and complemented strains at a multiplicity of infection (MOI) of 20. At 0.5 h.p.i. (hour post infection), 100 μg/mL gentamicin was added to each well to kill extracellular bacteria. At 0.5, 2, 4, and 8 h.p.i., the cells were lysed with sterile H_2_O containing 0.1% Triton X-100 for 15 min at room temperature. The diluted bacterial suspensions were plated onto a Hottinger’s agar to determine the number of viable bacteria as described above^32,48^.

### Assessment of virulence of *Y. pestis* to mice

Wide-type *Y. pestis* strain, mutants and complemented strains were grown separately in Luria-Bertani medium at 26 °C. For each group, 10–20 female BALB/c mice (8 weeks old) were infected subcutaneously at the dose of 100 CFU bacteria per mouse. The mice were observed for 14 days and the survival rates of each group were calculated as previously reported^46^. The survival rate of mice is equal to the remaining number of live animals divided by the initial number of animals in the group. The animal care was in accordance with institutional guidelines and ethical regulations of the Beijing Institute of Microbiology and Epidemiology.

### T3SS translocation assays

*Yersinia pestis* strains WT, ΔSEP-yp1, ΔSEP-yp2, and Δ*yopB* were grown at 26 °C in the TMH medium without calcium to an OD_620nm_ = 1.0, and then cultured for an additional 3 h at 37 °C to induce T3SS expression. HeLa cells (ATCC) were seeded into 24-well plates and grown to 80–90% confluence. HeLa cells were then infected with *Y. pestis* strains at an MOI of 10. After 2-h infection, HeLa cells were washed once in phosphate-buffered saline (PBS) and lysed for 15 min in lysis buffer containing 25 mM Tris-HCl (pH 7.6), 150 mM NaCl, 0.5% Triton X-100, and the protease inhibitor mixture. Cell lysates and pellets were separated by centrifugation. Equal amounts of lysates and pellets were separated by SDS-PAGE and transferred onto an Immobilon-P transfer membrane. Proteins were visualized using rabbit antibodies specific for recombinant YopE and YopM at a concentration of 1 µg/mL^44,49,50^.

### Quantitative analysis of the global proteome of *Y. pestis*

After *Y. pestis* strain, mutants and complemented strains were cultured, the bacteria obtained by centrifugation were resuspended and washed three times with cold PBS, and the resuspension was centrifuged again to obtain the bacteria pellets. Each group of samples was resuspended by adding four times the sample volume of lysis buffer containing 8 M urea, then sonicated on ice, and finally centrifuged at 20,000 × *g* for 10 min at 4 °C to remove residual cell debris. Protein concentration in lysates was measured using bicinchoninic acid protein assay. Proteins were reduced with 5 mM dithiothreitol at 56 °C for 30 min and alkylated with 10 mM iodoacetamide in the dark at room temperature for 30 min. After 4-fold dilution by 25 mM ammonium bicarbonate, proteins were digested by sequencing grade trypsin (1:50, w/w). The digestion was terminated by adding formic acid to pH 2. The solution was centrifuged to remove precipitates. Peptide mixtures were loaded on the Sep-Pak C18 cartridges (Waters) and eluted off by 50% acetonitrile with 0.1% formic acid, followed by drying down in a SpeedVac concentrator. The peptide samples were resuspended in water containing 0.1% formic acid and subjected to LC-MS analysis. All the parameters of LC-MS are the same as those for analysis of SEP digestion described above, except for the 120-min gradient: 4–8% solvent B for 5 min, 8–29% for 100 min, 29–43% for 11 min, 43–95% for 1 min, and 95% for 3 min (Solvent A, water; Solvent B, acetonitrile; 0.1% formic acid). The MS raw data were analyzed by PEAKS Studio v8.5 against a home-made *Y. pestis* protein database containing 4136 entries. Trypsin was set as an enzyme. Other search parameters are the same as those used for SEP described above. The procedures for label-free quantitative analysis and bioinformatic analysis are also the same.

The protein expression data were filtered to have at least 75% valid abundance values in all data. The missing values were imputed with 10% of the minimum values of the proteins. Standard statistical methods based on Student’s *t* test were used. Multiple hypothesis testing was controlled by using a Benjamini–Hochberg FDR threshold of 5%. Fold changes >2 or <0.5 and *p* value <0.05 in two comparable groups were considered to be significant. The unsupervised PCA model of proteome data was used in this study.

The Blast2GO (version 3.3.5) program^13^ was used to obtain GO annotations^51^. KEGG pathway^52^ online service tools KAAS were used to annotate protein’s KEGG database description. The STRING database (http://string-db.org)^53^ was used for predicting protein networks. All STRING network analyses were performed with Experimental and Databases evidence at a medium (0.4) confidence level. The networks were downloaded as tab-delimited text files and visualized and reorganized using the Cytoscape (3.2.1) software^54^.

### Statistics and reproducibility

For Fig. 4b–d, *p* values were calculated by unpaired *t* test. Three biological replicates were performed in each group. For Figs. 3a, 5c, and Supplementary Fig. 2. The *p* values were calculated by unpaired *t* test and adjusted with Benjamini–Hochberg procedure with FDR < 0.05. Three biological replicates were performed in each group. For Fig. 4e and Supplementary Fig. 4, log-rank test was used to calculate the *p* values. The *N* number is shown in the figure legends.

### Reporting summary

Further information on research design is available in the Nature Research Reporting Summary linked to this article.

## Supplementary information

Supplementary Information

Description of Additional Supplementary Files

Supplementary Data 1

Supplementary Data 2

Supplementary Data 3

Supplementary Data 4

Supplementary Data 5

Supplementary Data 6

Supplementary Data 7

Reporting Summary

## Data Availability

The mass spectrometry proteomics data have been deposited to the ProteomeXchange Consortium (http://proteomecentral.proteomexchange.org) through the iProX partner repository with the dataset identifier PXD028891. The RNA-seq data have been deposited in the National Center for Biotechnology Information (NCBI) database with accession code PRJNA662194. The Source data for graphs and charts is available as Supplementary Data 7. The uncropped immunoblot images of Figs. 3d, 5d, and 6 are shown in Supplementary Figs. 8, 9, and 10. Any remaining information can be obtained from the corresponding author upon reasonable request.

## Code availability

No custom algorithms or software were used in this work.
