# Supplementary material for: Proteogenomic discovery of sORF-encoded peptides associated with bacterial virulence in Yersinia pestis
Source: Commun Biol. 2021 Nov 2;4:1248. doi: 10.1038/s42003-021-02759-x (PMC8563848; doi:10.1038/s42003-021-02759-x)
Supplement: Supplementary file 1 — Supplementary Information [file 42003_2021_2759_MOESM1_ESM.pdf]

Supplementary information for:

Proteogenomic Discovery of sORF-encoded Peptides Associated with Bacterial Virulence in *Yersinia pestis*

Shiyang Cao[1,#], Xinyue Liu[2,3,#], Yin Huang[2,#], Yanfeng Yan[1,#], Congli Zhou, Chen Shao[2], Ruifu Yang[1], Weimin Zhu[2,*], Zongmin Du[1,*] and Chenxi Jia[2,*]

[1] State Key Laboratory of Pathogen and Biosecurity, Beijing Institute of Microbiology and Epidemiology, Beijing 100071, China

[2] State Key Laboratory of Proteomics, Beijing Proteome Research Center, Beijing Institute of Lifeomics, National Center for Protein Sciences (The PHOENIX Center, Beijing), Beijing 102206, China

[3] School of Life Sciences, Hebei University, Hebei Province, Baoding 071002, China

[#] These authors contribute equally to this work.

* Corresponding author

Correspondence: Prof. Chenxi Jia, Zongmin Du and Weimin Zhu

E-mail: cjia@mail.ncpsb.org.cn, zmduams@163.com, and wmzhuworld@gmail.com

Lead contact: Prof. Chenxi Jia,

Key words: Proteogenomics, mass spectrometry, microbiology, sORFs

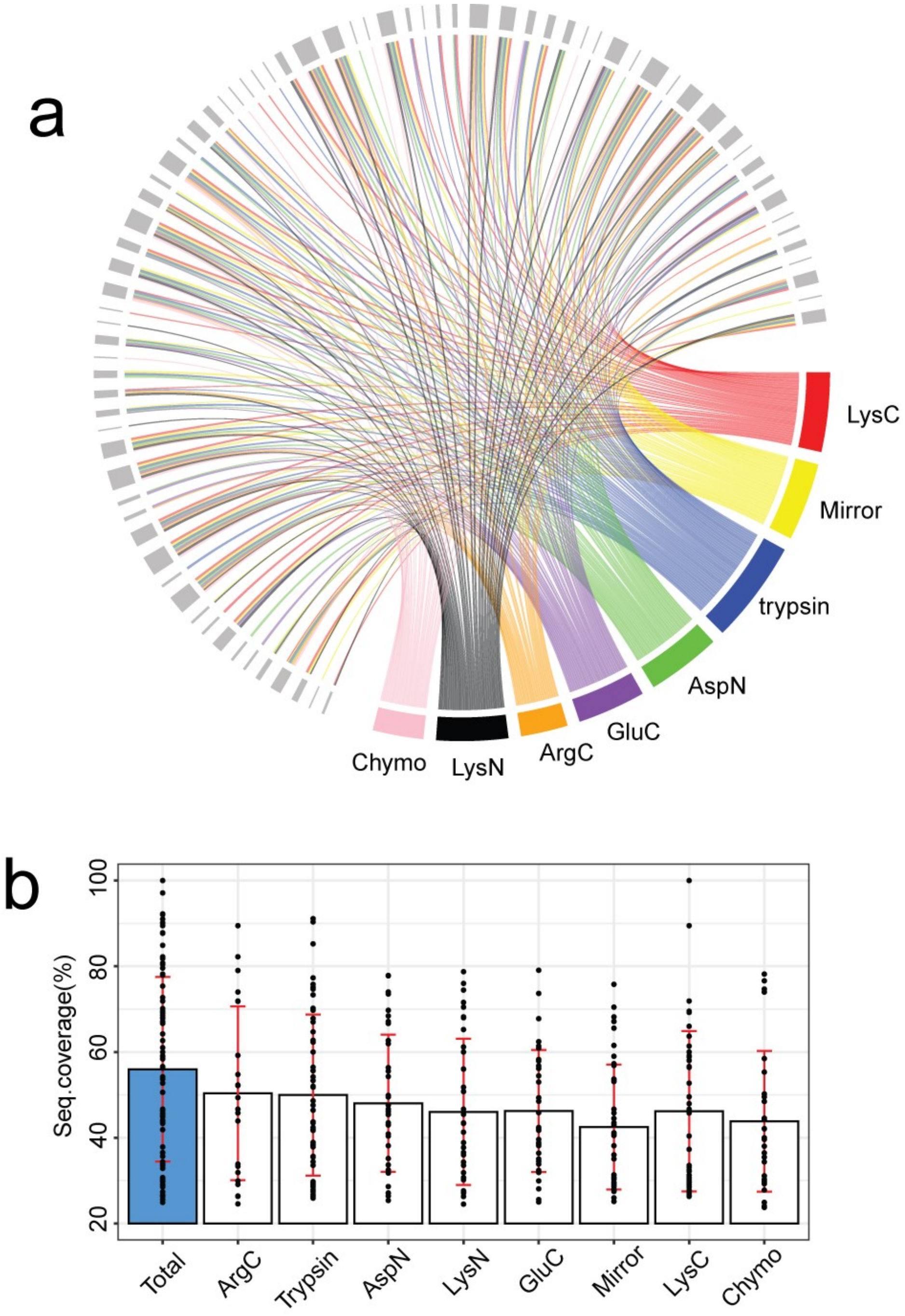

Supplementary Figure 1. Enhanced identifications by using multiple enzymes in SEP discovery. (a) Circos plot showing the distribution of the 76 identified SEPs in use of eight proteases. (b) Column scatter plot showing the enhanced sequence coverage due to use of eight proteases. Error bars represent standard deviation. Two biological replicates for each enzyme.

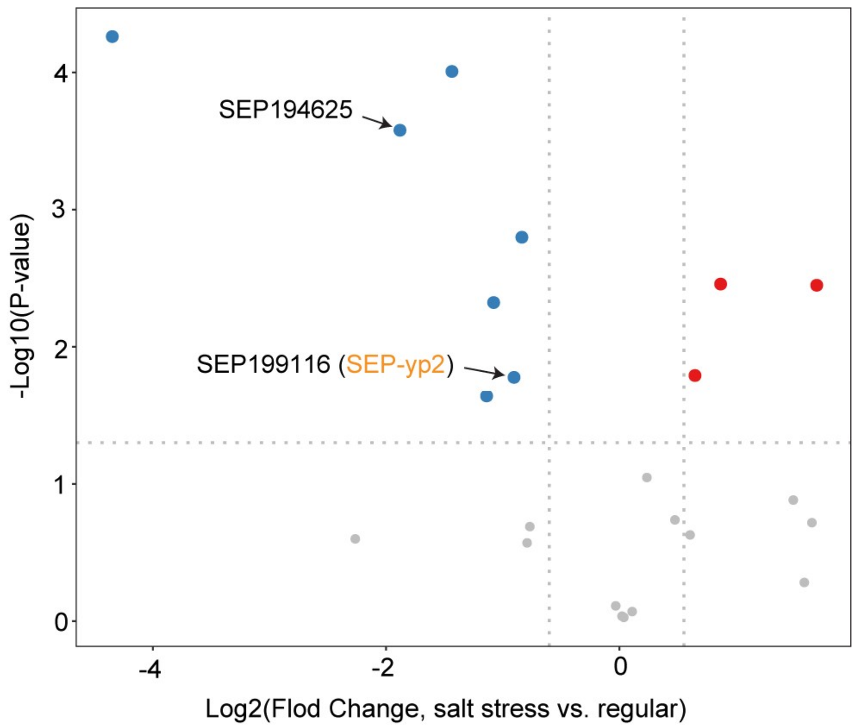

Supplementary Figure 2. Functional screening of SEPs under high-salinity stress by label-free quantitative peptidomics. Volcano plot showing the quantitative comparison between two culture conditions (26 °C in TMH without and with 2.5% NaCl). The *p*-value was calculated by unpaired t-test and adjusted with Benjamini-Hochberg procedure. The SEPs within top five abundance of transcripts (Figure 3B) was labeled. *N* = 3 in each group. Refer to Supplementary Data 4 for details.

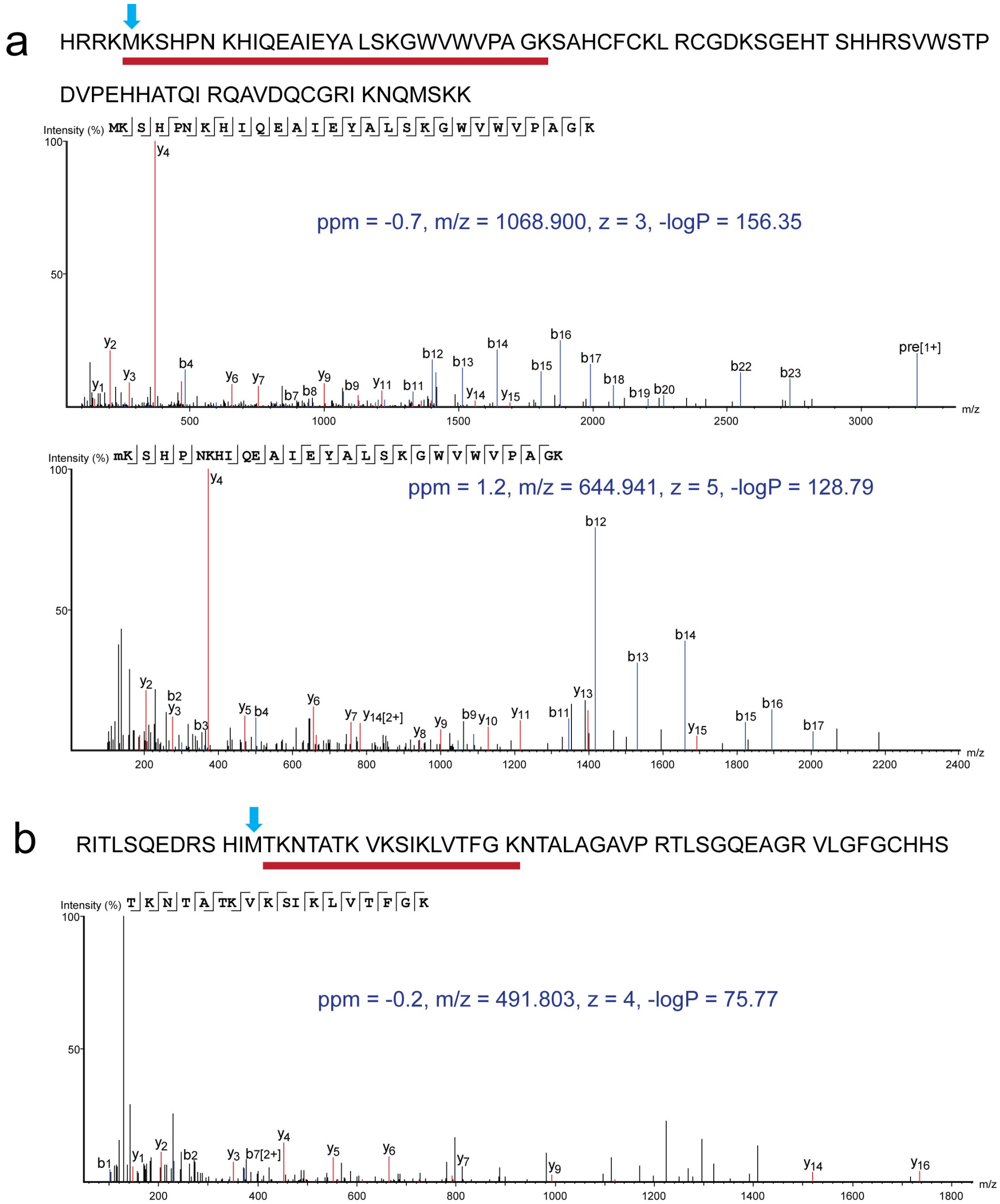

Supplementary Figure 3. Tandem mass spectrometry of unique peptides from N-terminus of SEP-yp2 (a) and -yp1 (b).

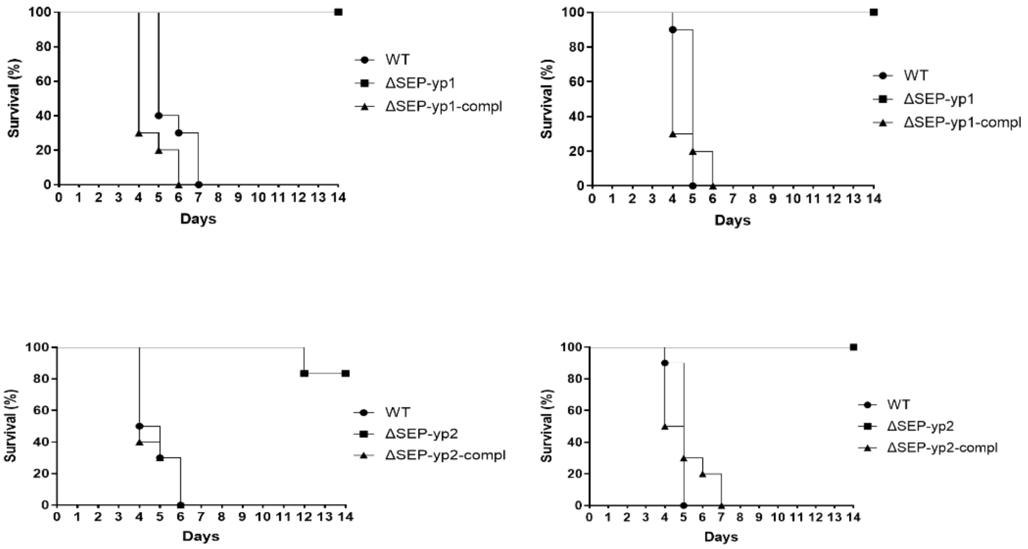

Replicate #2 **Replicate #3**

Supplementary Figure 4. Animal survival curve showing the bacterial virulence by subcutaneous injection to mice. Two replicates of the experiments are shown. WT, N=10; ΔSEP, N=10; and ΔSEP-compl, N=10. A logrank test was used to calculate the statistical significance. p-value (WT vs ΔSEP) < 0.005, and p-value (ΔSEP vs ΔSEP-compl) < 0.005.

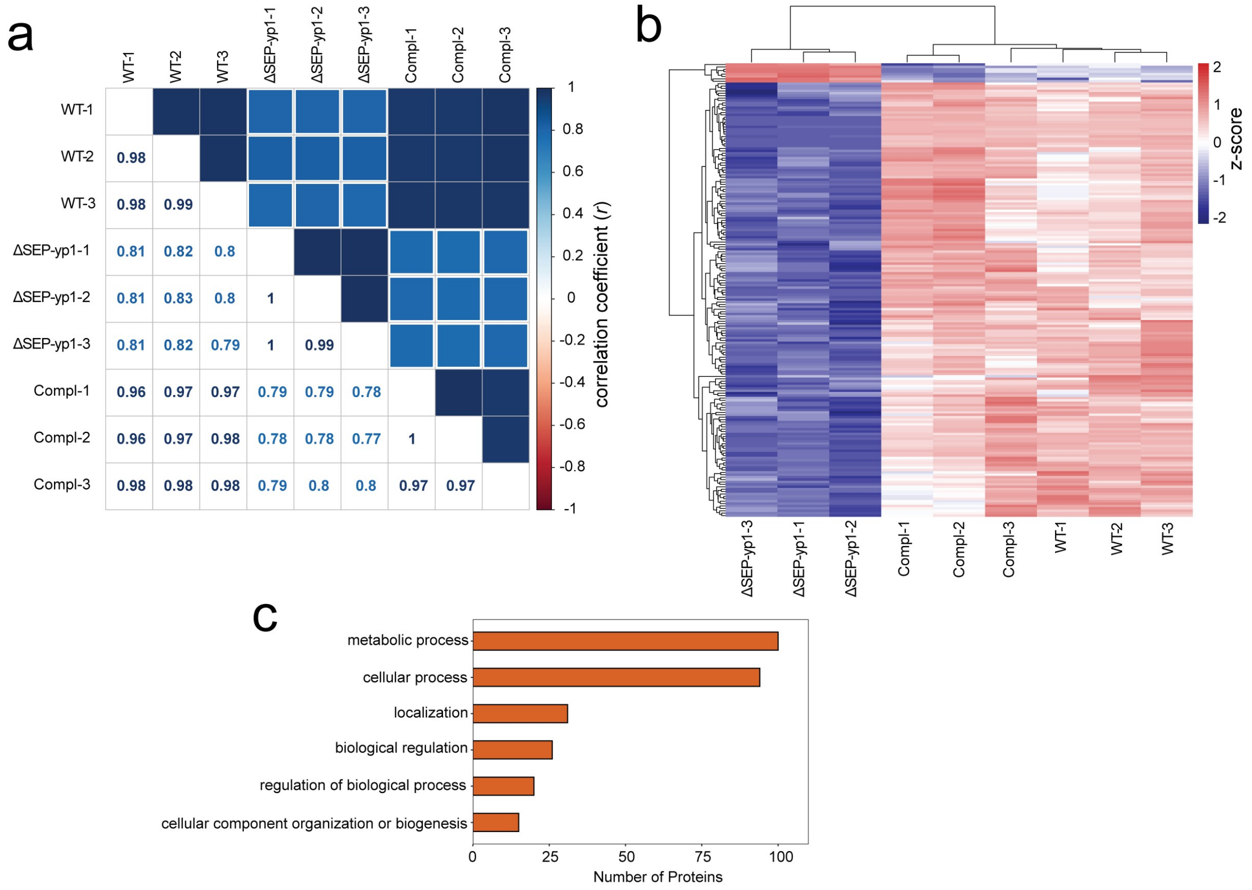

Supplementary Figure 5. Quantitative global proteome analysis of WT, ΔSEP-yp1 and ΔSEP-yp1-compl (compl). (a) Pearson correlation matrix of the nice samples in three groups. (b) Hierarchical clustering. (c) Gene ontology analysis. *N* = 3 in each group. Refer to Supplementary Data 5 for details.

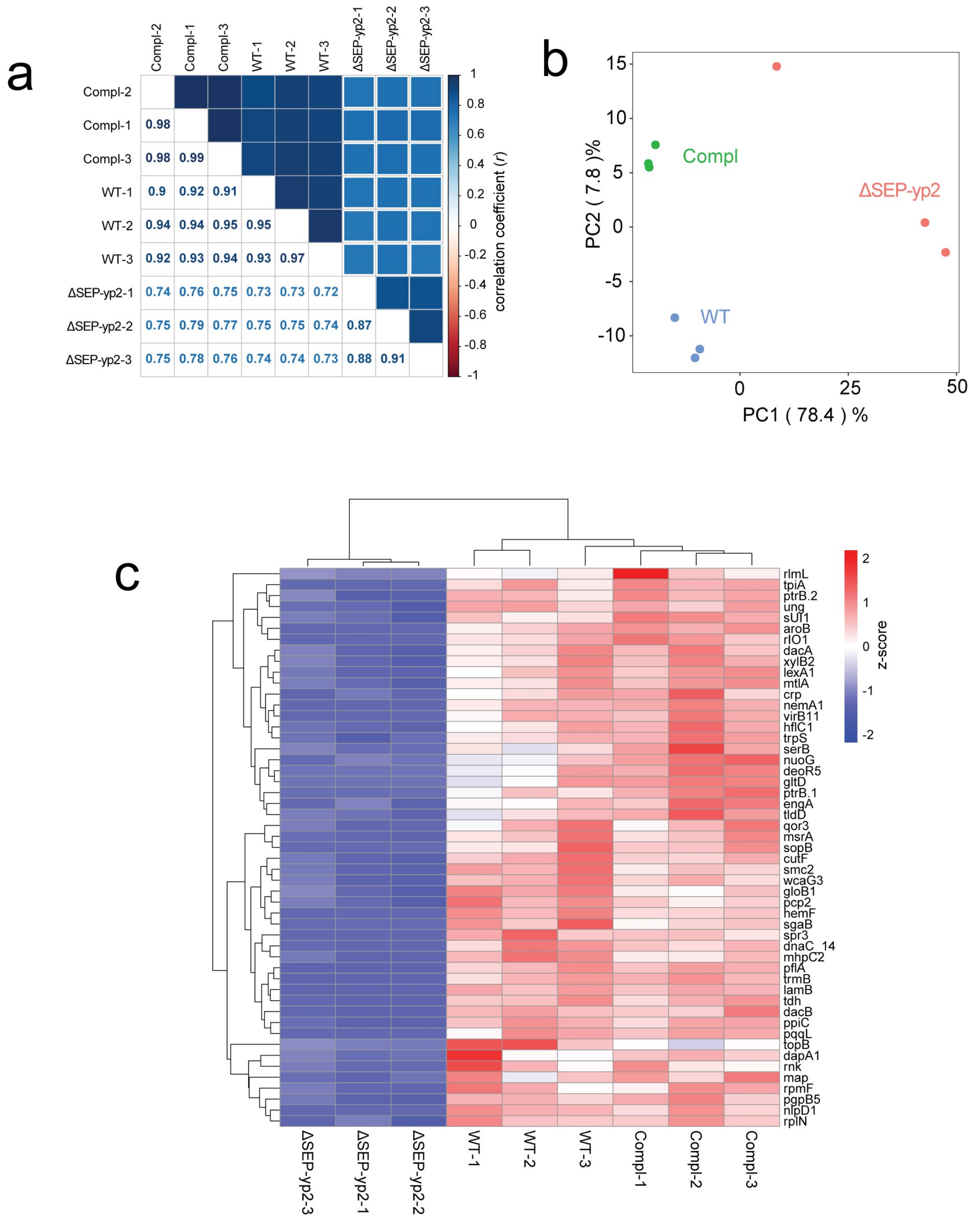

Supplementary Figure 6. Quantitative global proteome analysis of WT, ΔSEP-yp2 and ΔSEP-yp2-compl (compl). (a) Pearson correlation matrix of the nine samples in three groups. (b) Principal component analysis. (c) Hierarchical clustering. *N* = 3 in each group. Refer to Supplementary Data 6 for details.

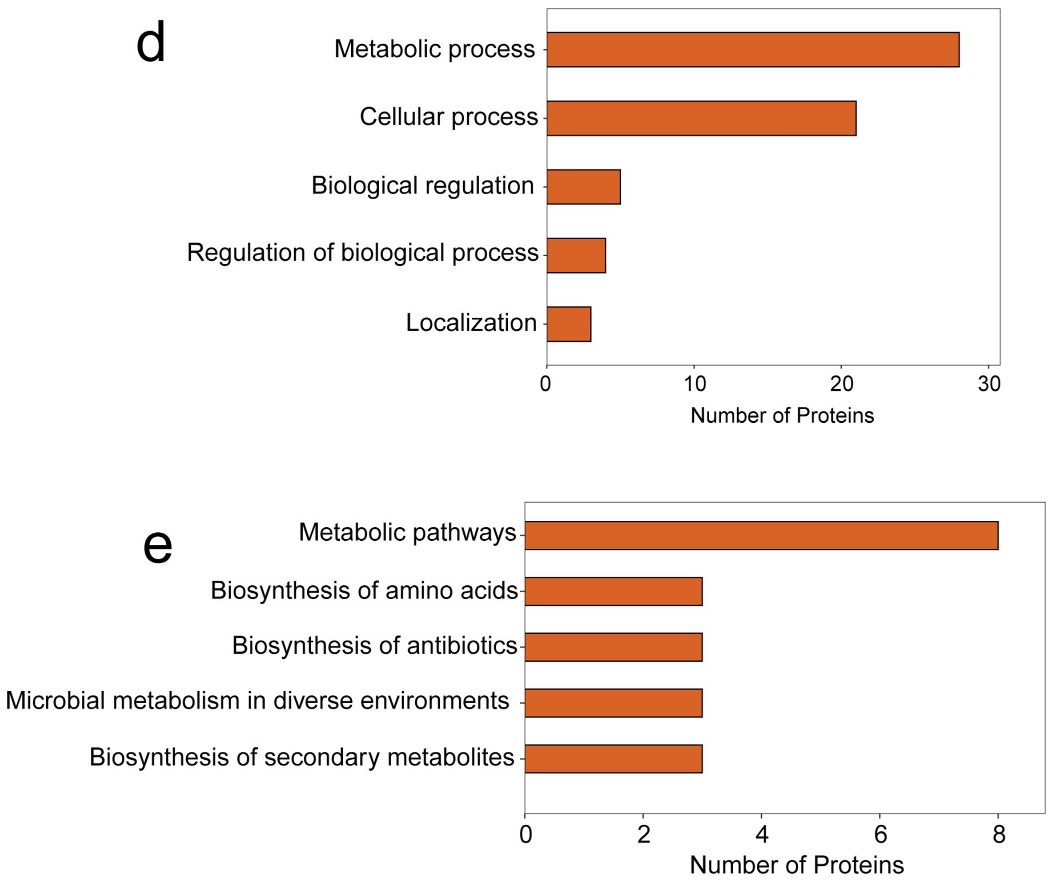

Continued Supplementary Figure 6. (d) Gene ontology analysis. (e) KEGG analysis. $N = 3$ in each group. Refer to Supplementary Data 6 for details.

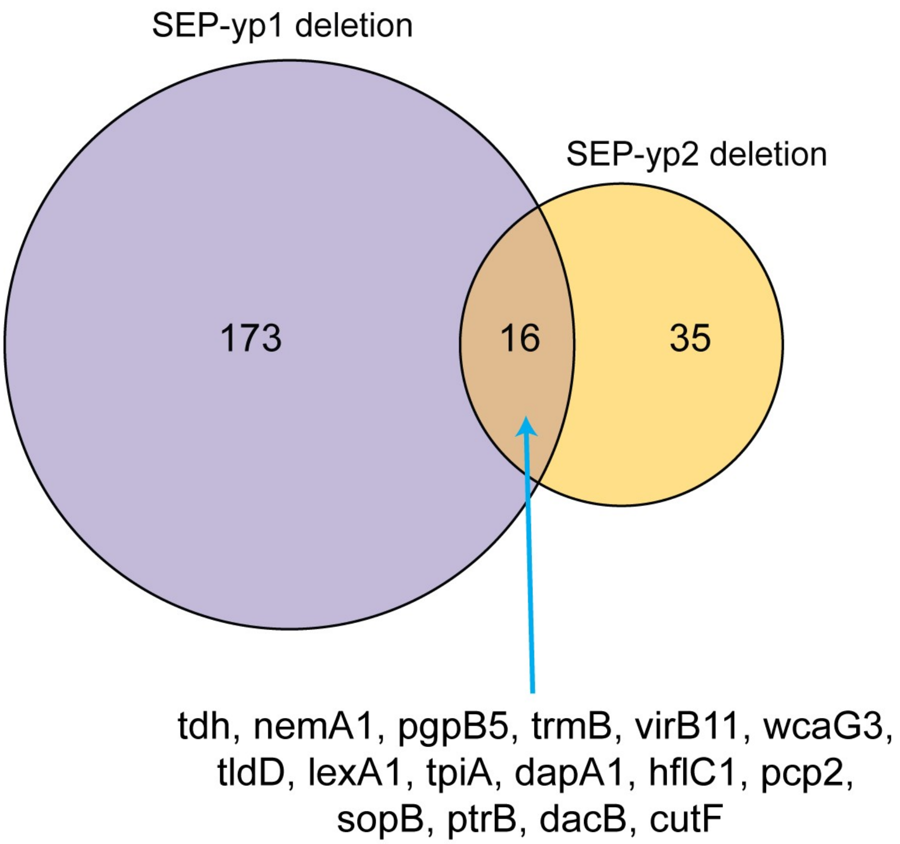

Supplementary Figure 7. Venn diagram showing the altered proteins in *Y. pestis* by deletion of SEP-yp1 and SEP-yp2.

Figure 3D, ΔSEP-yp2

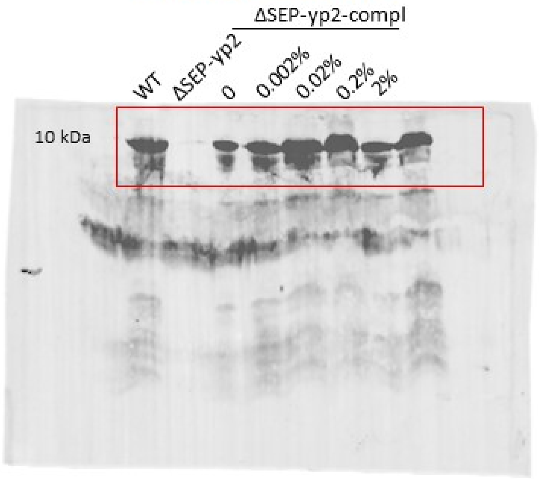

Figure 3D, ΔSEP-yp1

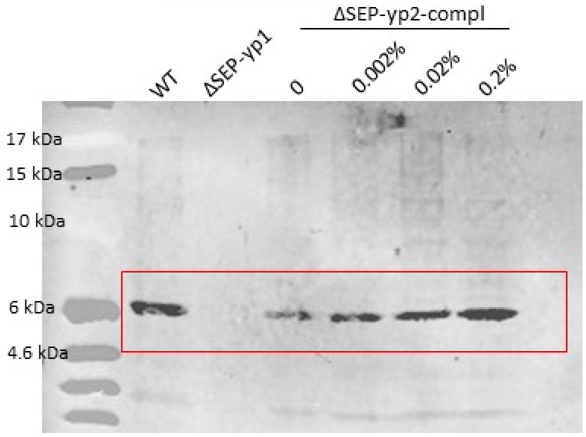

Supplementary Figure 8. Uncropped immunoblot images of SEP-yp2 and SEP-yp1 showing bacterial lysis of *Y. pestis* WT, ΔSEP and ΔSEP-compl. These images correspond to Figure 3D.

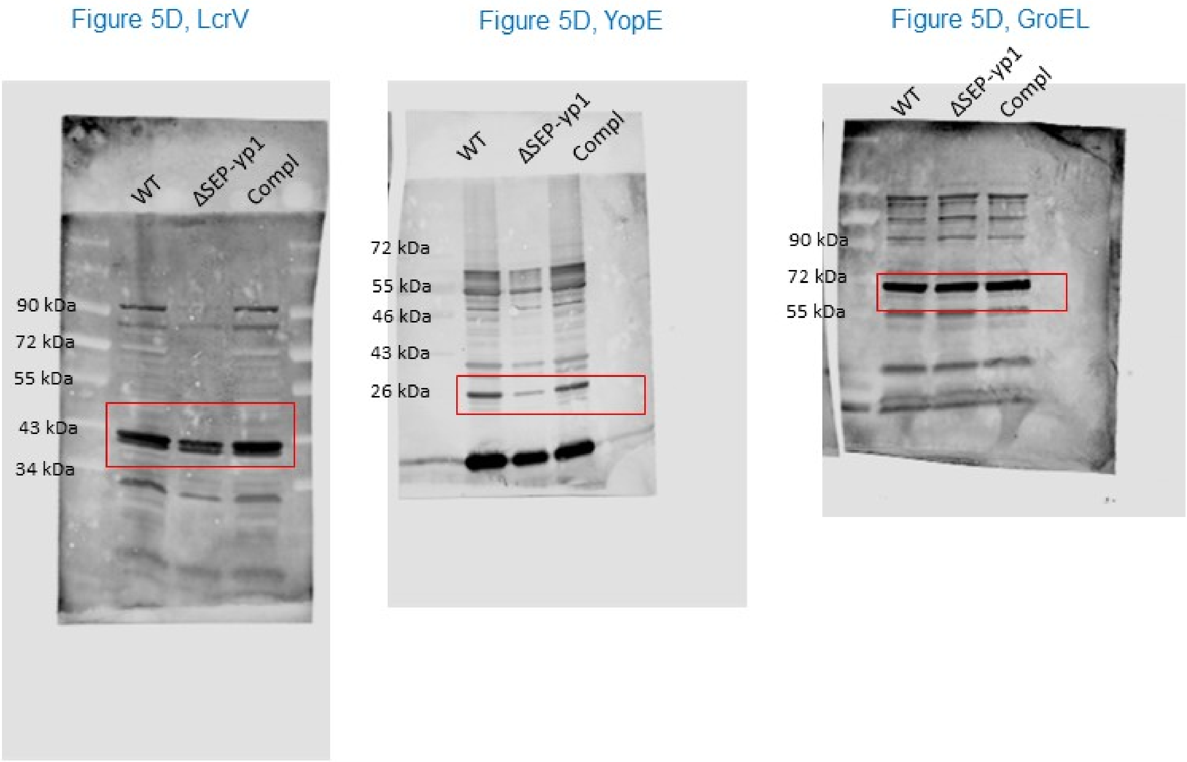

Supplementary Figure 9. Uncropped immunoblot images of the orthogonal validation of the altered proteins. These images correspond to Figure 5D.

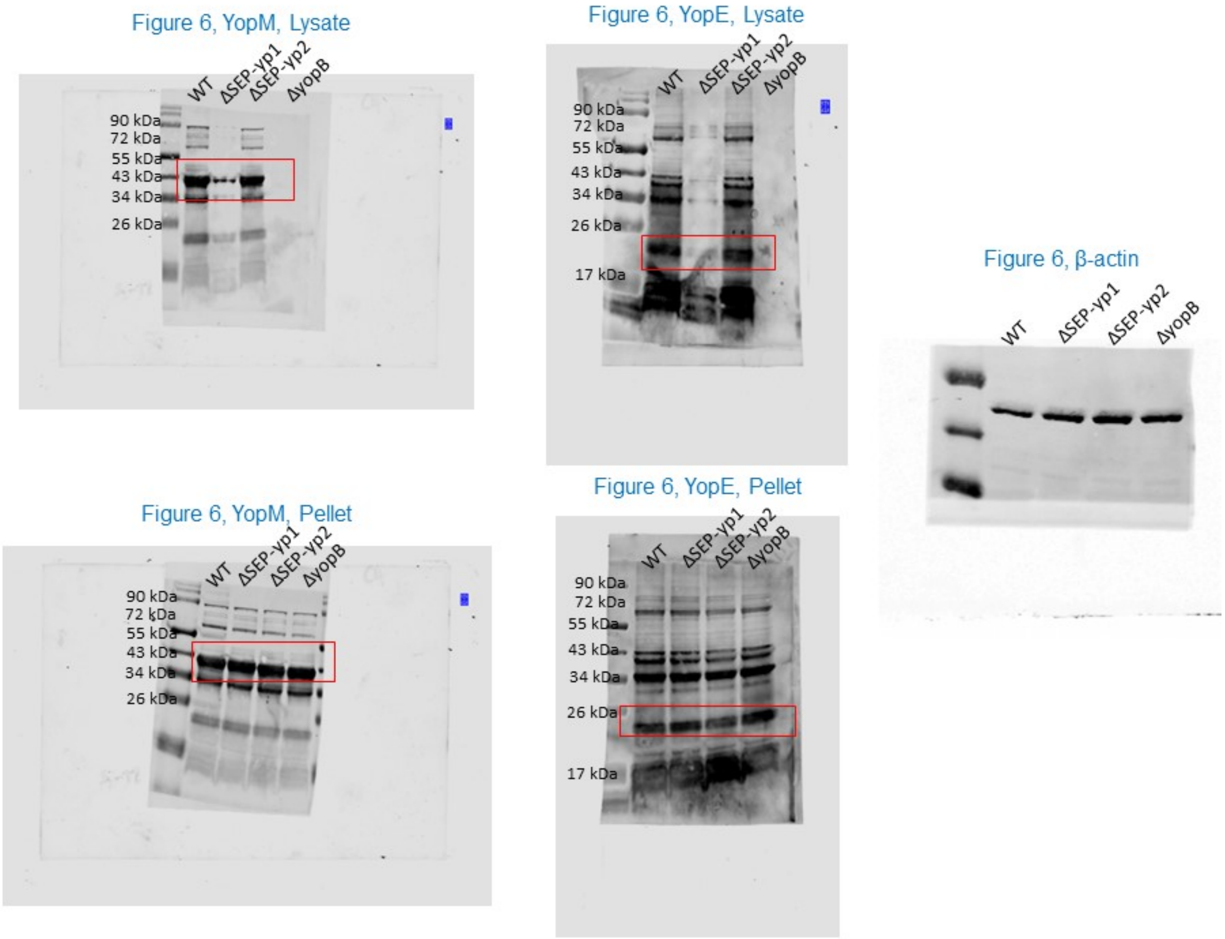

Supplementary Figure 10. Uncropped immunoblot images showing that deletion of SEP-yp1 influences the translocation of T3SS. These images correspond to Figure 6.

Supplementary Table 1. Plasmids and bacterial strains used in this study.

Strains or plasmids	Descriptions	Sources
**Plasmids**		
pKD46	Temperature-sensitive plasmid expressing λRed recombinase under the control of arabinose; Apr	Laboratory collection
pBAD24	Plasmids for expression of the cloned gene under the control of arabinose; Apr	Laboratory collection
pBAD24-SEP-yp1	SEP-yp1 gene was inserted into pBAD24; Ap^r	This study
pBAD24-SEP-yp2	SEP-yp2 gene was inserted into pBAD24	This study
**Strains**		
201 strain	Wild-type *Y. pestis* strain	
201-pKD46	201 strain containing plasmid pKD46	This study
ΔSEP-yp1	SEP-yp1 gene was replaced by Kanamycin cassette	This study
ΔSEP-yp2	SEP-yp2 gene was replaced by Kanamycin cassette	This study
201-pBAD24-SEP-yp1	201 strain containing plasmid pBAD24-SEP-yp1	This study
201-pBAD24-SEP-yp2	201 strain containing plasmid pBAD24-SEP-yp2	This study
Δ*yopB*	The *yopB* gene was replaced by Kanamycin cassette	This study
