# Supplementary material for: Proteogenomic discovery of sORF-encoded peptides associated with bacterial virulence in Yersinia pestis
Source: Commun Biol. 2021 Nov 2;4:1248. doi: 10.1038/s42003-021-02759-x (PMC8563848; doi:10.1038/s42003-021-02759-x)
Supplement: Supplementary file 2 — Description of Additional Supplementary Files [file 42003_2021_2759_MOESM2_ESM.pdf]

Description of Additional Supplementary Files

File name: Supplementary Data 1.
Description: The detailed information of the SEP candidates filtered at three stages. Refer to Figure 1 in the main text for the filtering criteria.

File name: Supplementary Data 2.
Description: Determination of the N-terminus of the identified *Y. pestis* SEPs.

File name: Supplementary Data 3.
Description: Bioinformatic analysis of the identified 76 SEPs. The four sheets correspond to Figure 2B, 2C, 2D and 2E, respectively.

File name: Supplementary Data 4.
Description: Detailed information for quantitative peptidomics analysis of SEPs and accumulative intensities of SEPs in transcriptome. The three sheets correspond to Figure 3A, Supplementary Figure 2 and Figure 3B, respectively.

File name: Supplementary Data 5.
Description: The detailed information of quantitative global proteome analysis of WT, ΔSEP-yp1 and ΔSEP-yp1-compl (compl) . These information corresponds to Figure 5 in the main text.

File name: Supplementary Data 6.
Description: The detailed information of quantitative global proteome analysis of WT, ΔSEP-yp2 and ΔSEP-yp2-compl (compl).

File name: Supplementary Data 7.
Description: Source data underlying the graphs and charts.
