# Supplementary material for: Proteogenomic discovery of sORF-encoded peptides associated with bacterial virulence in Yersinia pestis
Source: Commun Biol. 2021 Nov 2;4:1248. doi: 10.1038/s42003-021-02759-x (PMC8563848; doi:10.1038/s42003-021-02759-x)
Supplement: Supplementary file 10 — Reporting Summary [file 42003_2021_2759_MOESM10_ESM.pdf]

Corresponding author(s): Chenxi Jia
Last updated by author(s): Sep 11, 2021

Reporting Summary

Nature Research wishes to improve the reproducibility of the work that we publish. This form provides structure for consistency and transparency in reporting. For further information on Nature Research policies, see our Editorial Policies and the Editorial Policy Checklist.

Statistics

For all statistical analyses, confirm that the following items are present in the figure legend, table legend, main text, or Methods section.

n/a	Confirmed	
☐	☒	The exact sample size (*n*) for each experimental group/condition, given as a discrete number and unit of measurement
☐	☒	A statement on whether measurements were taken from distinct samples or whether the same sample was measured repeatedly
☐	☒	The statistical test(s) used AND whether they are one- or two-sided<br>*Only common tests should be described solely by name; describe more complex techniques in the Methods section.*
☒	☐	A description of all covariates tested
☐	☒	A description of any assumptions or corrections, such as tests of normality and adjustment for multiple comparisons
☐	☒	A full description of the statistical parameters including central tendency (e.g. means) or other basic estimates (e.g. regression coefficient) AND variation (e.g. standard deviation) or associated estimates of uncertainty (e.g. confidence intervals)
☒	☐	For null hypothesis testing, the test statistic (e.g. *F*, *t*, *r*) with confidence intervals, effect sizes, degrees of freedom and *P* value noted<br>*Give P values as exact values whenever suitable.*
☒	☐	For Bayesian analysis, information on the choice of priors and Markov chain Monte Carlo settings
☒	☐	For hierarchical and complex designs, identification of the appropriate level for tests and full reporting of outcomes
☒	☐	Estimates of effect sizes (e.g. Cohen's *d*, Pearson's *r*), indicating how they were calculated

Our web collection on statistics for biologists contains articles on many of the points above.

Software and code

Policy information about availability of computer code

Data collection	N/A
Data analysis	N/A

For manuscripts utilizing custom algorithms or software that are central to the research but not yet described in published literature, software must be made available to editors and reviewers. We strongly encourage code deposition in a community repository (e.g. GitHub). See the Nature Research guidelines for submitting code & software for further information.

Data

Policy information about availability of data

All manuscripts must include a data availability statement. This statement should provide the following information, where applicable:

- Accession codes, unique identifiers, or web links for publicly available datasets
- A list of figures that have associated raw data
- A description of any restrictions on data availability

The mass spectrometry proteomics data have been deposited to the ProteomeXchange Consortium (http://proteomecentral.proteomexchange.org) through the iProX partner repository with the dataset identifier PXD015323. The RNAseq data have been deposited in the National Center for National Center for Biotechnology Information (NCBI) database with accession code PRJNA662194.

Field-specific reporting

Please select the one below that is the best fit for your research. If you are not sure, read the appropriate sections before making your selection.

☒ Life sciences ☐ Behavioural & social sciences ☐ Ecological, evolutionary & environmental sciences

For a reference copy of the document with all sections, see nature.com/documents/nr-reporting-summary-flat.pdf

Life sciences study design

All studies must disclose on these points even when the disclosure is negative.

Sample size	For identification of SEPs, 16 samples (equal to 16 biological replicates) were respectively digested using eight enzymes followed by further analysis. For quantitative analysis, each group contains three or more samples.
Data exclusions	No data was excluded.
Replication	At least three biological replicates were performed.
Randomization	The samples were manually selected into each group in a random manner. The samples were randomly analyzed to minimize the batch effect.
Blinding	The researchers between data acquisition and data analysis were blinded from each other.

Reporting for specific materials, systems and methods

We require information from authors about some types of materials, experimental systems and methods used in many studies. Here, indicate whether each material, system or method listed is relevant to your study. If you are not sure if a list item applies to your research, read the appropriate section before selecting a response.

Materials & experimental systems

n/a	Involved in the study	
☐	☒	Antibodies
☒	☐	Eukaryotic cell lines
☒	☐	Palaeontology and archaeology
☐	☒	Animals and other organisms
☒	☐	Human research participants
☒	☐	Clinical data
☒	☐	Dual use research of concern

Methods

n/a	Involved in the study	
☒	☐	ChIP-seq
☒	☐	Flow cytometry
☒	☐	MRI-based neuroimaging

Antibodies

Antibodies used	Antibodies for SEPs-yp1, SEPs-yp2, LcrV, YopE were home-made.
Validation	Western blot experiments were performed on wild strains and gene-deleted strains, combined with protein molecular weight, to validate antibody.

Animals and other organisms

Policy information about studies involving animals; ARRIVE guidelines recommended for reporting animal research

Laboratory animals	Bal B/C mice aged 6-8 weeks were SPF grade, and they were taken out after one week of acclimatization in the animal room.
Wild animals	not involved
Field-collected samples	not involved
Ethics oversight	The Y. pestis virulence experiment on mice was carried out in the P3 laboratory at Beijing Institute of Microbiology and Epidemiology. The relevant ethics number of this article was: IACUC-DWZX-2020-071.

Note that full information on the approval of the study protocol must also be provided in the manuscript.
